# Iron deficiency anemia is associated with renal function decline in obstructive sleep apnea: a multi-institutional cohort study

**DOI:** 10.3389/fnut.2026.1743873

**Published:** 2026-02-12

**Authors:** Kuo-Chuan Hung, Hsiu-Lan Weng, Yao-Tsung Lin, Chun-Ning Ho, Jheng-Yan Wu, Pei-Han Fu, I-Wen Chen

**Affiliations:** 1 Department of Anesthesiology, Chi Mei Medical Center, Tainan City, Taiwan; 2 School of Medicine, College of Medicine, National Sun Yat-sen University, Kaohsiung, Taiwan; 3 Department of Anesthesiology, E-DA Hospital, I-Shou University, Kaohsiung City, Taiwan; 4 Department of Nutrition, Chi Mei Medical Center, Tainan City, Taiwan; 5 Department of Anesthesiology, Chi Mei Medical Center, Liouying, Tainan City, Taiwan

**Keywords:** acute kidney injury, chronic kidney disease, iron deficiency anemia, obstructive sleep apnea, propensity score matching, renal function decline

## Abstract

**Background:**

Obstructive sleep apnea (OSA) is associated with an increased risk of chronic kidney disease through mechanisms including intermittent hypoxia and systemic inflammation. Iron deficiency anemia (IDA) may synergistically worsen renal vulnerability in patients with OSA through overlapping hypoxic and oxidative pathways; however, this relationship remains poorly characterized.

**Methods:**

This retrospective cohort study utilized the TriNetX Research Network to identify and analyze adult patients with a first diagnosis of obstructive sleep apnea (OSA) between 2010 and 2022. After propensity score matching, 38,064 patients with pre-existing IDA were compared with 38,064 matched controls without IDA. The primary outcome was the 5-year cumulative incidence of composite renal function decline, defined as progression to chronic kidney disease stage 4 or 5, end-stage renal disease, or hemodialysis initiation. Secondary outcomes included acute kidney injury (AKI), pulmonary hypertension, all-cause mortality, and intensive care unit (ICU) admission.

**Results:**

At five-year follow-up, IDA was associated with increased cumulative incidence of composite renal function decline compared to controls [2.0% versus 1.6%; hazard ratio (HR): 1.23, 95% confidence interval (CI):1.10–1.37, *p* < 0.001]. Significant associations were also observed for AKI (10.7% vs. 8.6%; HR: 1.22, *p* < 0.001), pulmonary hypertension (4.5% vs. 3.7%; HR:1.22, *p* < 0.001), all-cause mortality (6.4% vs. 5.0%; HR:1.29, *p* < 0.001), and ICU admission (6.9% vs. 6.0%; HR:1.17, *p* < 0.001). These associations persisted at seven-year follow-up and across sensitivity analyses.

**Conclusion:**

IDA is associated with an increased risk of renal function decline and adverse clinical outcomes in patients with OSA, suggesting a potentially modifiable risk factor that warrants further investigation. Given the retrospective design and reliance on electronic health record data, prospective studies are required to confirm these findings and elucidate the underlying mechanisms.

## 1 Introduction

Obstructive sleep apnea (OSA) affects approximately 34% of middle-aged men and 17% of women, representing a major public health concern (1). This highly prevalent disorder is characterized by recurrent episodes of upper airway obstruction during sleep, leading to intermittent hypoxia, sleep fragmentation, and sympathetic activation (2, 3). These pathophysiological disturbances contribute to a wide range of adverse health outcomes, including hypertension, arrhythmia, coronary artery disease, stroke, insulin resistance, and dyslipidemia (4–7). Beyond its well-established effects on cardiovascular health, emerging evidence links OSA to progressive kidney disease through mechanisms such as chronic intermittent hypoxia, sympathetic activation, and systemic inflammation (8–11). Population-based studies have demonstrated that patients with OSA face an increased risk of incident chronic kidney disease (CKD) and accelerated decline in renal function independent of traditional risk factors such as hypertension and diabetes mellitus (12–15).

Iron deficiency anemia (IDA) is among the most common nutritional disorders globally and disproportionately affects women owing to menstruation, pregnancy, and dietary factors (16). Disruptions in iron homeostasis can contribute to renal injury through mitochondrial dysfunction, oxidative stress, and endothelial impairment (17, 18). Experimental studies further demonstrated that iron deficiency amplifies kidney damage by enhancing oxidative injury and ferroptosis, thereby accelerating tubular and vascular injury (19). Clinically, reduced hemoglobin levels or anemia have been identified as independent predictors of rapid renal function decline in patients with type 2 diabetes, underscoring the importance of adequate oxygen delivery and mitochondrial efficiency for kidney health (20, 21). Despite this growing evidence, most investigations have focused on the general or diabetic population, while limited research has explored these associations in individuals with OSA. Given that OSA induces chronic intermittent hypoxia, sympathetic activation, and systemic inflammation, the coexistence of IDA may synergistically worsen renal vulnerability by overlapping the hypoxic and oxidative pathways. Accordingly, this study aimed to examine the association between IDA and renal function decline in a large, multi-institutional cohort of adults with a first diagnosis of OSA.

## 2 Methods

### 2.1 Study design and data source

This retrospective cohort study was conducted using the TriNetX Research Network, a federated health research platform that enables real-time analysis of de-identified electronic health record data contributed by more than 150 healthcare organizations throughout the United States. The network encompasses anonymized patient-level information, including demographics, diagnoses, procedures, laboratory results, and medication prescriptions. Each participating institution contributes internally validated data that undergo quality assurance processes to ensure accuracy and completeness. The TriNetX Research Network has been used in prior peer-reviewed observational and outcomes research studies across multiple clinical domains (22–24). The study protocol was reviewed and approved by the Institutional Review Board, which waived the requirement for informed consent in accordance with the regulations governing retrospective research using de-identified data.

### 2.2 Study population

Adult patients with a first diagnosis of OSA between 2010 and 2022 were included, and the diagnosis date was defined as the index date. Patients were classified into the IDA group if they had a documented history of IDA prior to the index date, whereas those without such a history constituted the control group. To ensure adequate data capture and minimize loss to follow-up, patients were required to have at least one healthcare encounter within 3 years preceding the index date and at least one visit occurring between 3 months and 5 years after the index date.

The following exclusion criteria were applied to ensure cohort consistency: presence of other anemia types including vitamin B12 deficiency anemia, folate deficiency anemia, other nutritional anemias, anemia associated with chronic disease, or other unclassified anemias; advanced renal disease defined as stage 4 or 5 CKD, end-stage renal disease, dialysis requirement, or estimated glomerular filtration rate below 30 mL/min/1.73 m^2^; history of major surgical procedures potentially affecting renal function or nutritional absorption including bariatric surgery, nephrectomy, or kidney transplantation; presence of polycystic kidney disease; or history of pulmonary hypertension. The detailed International Classification of Diseases, Tenth Revision, Clinical Modification (ICD-10-CM) codes used to define obstructive sleep apnea and iron deficiency anemia, as well as codes for exclusion criteria, outcome definitions, and variables included in propensity score matching, are provided in Supplemental Table 1.

### 2.3 Baseline characteristics and propensity score matching

Patient characteristics were systematically assessed during the three-year period preceding the index date. Demographic factors, including age, sex, and race were extracted from electronic health records. Comorbid diseases such as hypertension, cardiovascular disease, obesity, dyslipidemia, and diabetes mellitus were identified using ICD-10-CM codes. Laboratory parameters, such as serum albumin, estimated glomerular filtration rate, and hemoglobin A1c levels, were recorded when available. Medication profiles were comprehensively captured, with particular attention paid to agents that potentially influence renal function, such as angiotensin-converting enzyme inhibitors, angiotensin II receptor, and sodium-glucose cotransporter-2 inhibitors.

To minimize confounding and approximate randomized treatment allocation, propensity score matching was conducted using multivariable logistic regression that included all available demographic variables, comorbidities, laboratory parameters, and medication exposure. One-to-one greedy nearest-neighbor matching without replacement was applied with a caliper width of 0.1 standard deviations of the logit of the propensity score. Covariate balance between the matched cohorts was assessed using standardized mean differences, with values <0.1 indicating adequate balance. Covariate balance before and after propensity score matching was additionally assessed using a Love plot displaying standardized mean differences for all matching variables.

### 2.4 Outcome assessment

The primary outcome was cumulative incidence of composite renal function decline at five-year follow-up, defined as the development of CKD stage 4 or 5, end-stage renal disease, or initiation of hemodialysis. The secondary outcomes included cumulative incidence of incident acute kidney injury (AKI), incident pulmonary hypertension, all-cause mortality, and intensive care unit (ICU) admission. To establish an appropriate temporal sequence and minimize reverse causation bias, a three-month lag period was implemented, with all outcomes assessed beginning 3 months after the index date (i.e., initial diagnosis of OSA). Temporal trends were additionally explored by evaluating outcomes at the 7-year follow-up.

### 2.5 Sensitivity analyses and subgroup analysis

Sensitivity analyses were conducted to test the robustness of findings across alternative analytical assumptions. Model I restricted the cohort to patients diagnosed between 2016 and 2020 to examine consistency using more recent data while ensuring that all included patients, including those diagnosed in 2020, had the opportunity for complete five-year follow-up. Model II excluded all patients with any baseline CKD history (i.e., CKD stage 1–5) to assess the associations in individuals with normal renal function at baseline. Subgroup analyses stratified by sex, age (18–50 vs. >50 years), hypertension status, obesity, cardiovascular disease, and diabetes mellitus were performed to explore potential effect modifications. The propensity score matching approach was applied identically within each sensitivity and subgroup analysis.

### 2.6 Statistical analysis

In the TriNetX database, missing data were handled using an available-case analysis approach without imputation. Laboratory values and clinical measurements were considered missing if they were not recorded in the electronic health records during the observation period. For propensity score estimation, only patients with available data for matching variables were included in the model, and no imputation methods were applied to fill in the missing values. This approach reflects real-world clinical practice, where the absence of recorded measurements often indicates that such tests were not clinically indicated or performed. Baseline characteristics were analyzed using only the available data. This strategy was adopted to maintain the integrity of the observed clinical data and avoid introducing potential bias from imputation assumptions in this large-scale observational study.

Descriptive statistics were used to summarize the baseline characteristics, with continuous variables presented as means with standard deviations and categorical variables as counts with percentages. Time-to-event outcomes were analyzed using Cox proportional hazards regression stratified by matched pairs, yielding hazard ratios (HRs) with 95% confidence intervals (CIs). The proportional hazards assumption was verified using Schoenfeld residual testing, and survival differences were compared using log-rank tests. Statistical significance was determined using a two-tailed alpha level of 0.05. All analyses were performed using the integrated analytical functions of the TriNetX platform.

## 3 Results

### 3.1 Patient selection and baseline characteristics

**Figure 1:**
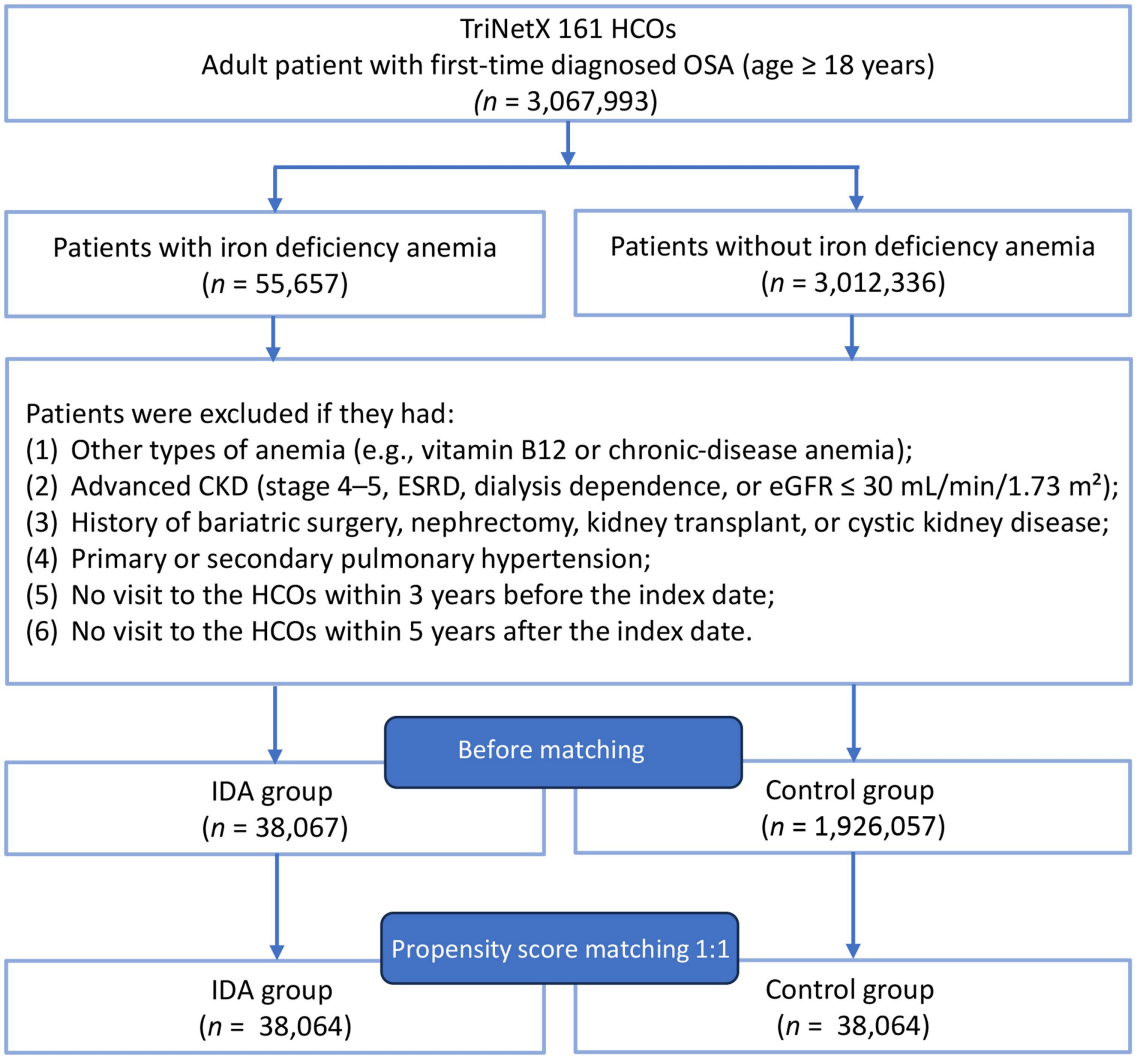
Patient selection flowchart from the TriNetX database. HCOs, Healthcare Organizations; CKD, Chronic kidney disease; ESRD, End-stage renal disease; eGFR, Estimated glomerular filtration rate; IDA, Iron deficiency anemia.

Between 2010 and 2022, data were retrieved from the TriNetX Research Network (Figure 1). After applying the predefined inclusion and exclusion criteria, 38,067 patients with IDA and 1,926,057 patients without IDA were identified. Propensity score matching yielded 38,064 matched pairs with an adequate covariate balance. Before matching, patients with IDA demonstrated a higher prevalence of most comorbidities compared to controls, with standardized mean differences exceeding 0.1 for multiple variables (Table 1). After matching, all baseline characteristics achieved a balance between the groups, with standardized mean differences below 0.1 (Supplemental Figure 1). The matched cohorts had a mean age of 54.0 ± 16.4 years in the IDA group and 53.8 ± 16.3 years in the control group. Female patients comprised 69.2 and 71.3% of patients in the IDA and control groups, respectively. Common comorbidities included essential hypertension (54.1% vs. 54.2%), dyslipidemia (44.4% vs. 44.3%), and overweight and obesity (41.2% vs. 41.6%) in the IDA and control groups, respectively.

**Table 1:** Baseline characteristics of patients with obstructive sleep apnea before and after propensity score matching.

Variables	Before matching			After matching		
	IDA group (*n* = 38,067)	Control group (*n* = 1,926,057)	SMD†	IDA group (*n* = 38,064)	Control group (*n* = 38,064)	SMD†
Patient characteristics						
Age at index (years)	54.0 ± 16.4	53.5 ± 16.6	0.026	54.0 ± 16.4	53.8 ± 16.3	0.010
Female	26,338 (69.2)	822,985 (42.7)	0.553	26,335 (69.2)	27,137 (71.3)	0.046
BMI ≥ 30 kg/m^2^	20,864 (54.8)	837,200 (43.5)	0.228	20,861 (54.8)	21,105 (55.4)	0.013
White	24,777 (65.1)	1,416,809 (73.6)	0.184	24,776 (65.1)	24,904 (65.4)	0.007
Black or African American	8,226 (21.6)	247,835 (12.9)	0.233	8,224 (21.6)	8,303 (21.8)	0.005
Other Race	1,268 (3.3)	58,483 (3.0)	0.017	1,268 (3.3)	1,193 (3.1)	0.011
Asian	755 (2.0)	48,785 (2.5)	0.037	755 (2.0)	721 (1.9)	0.006
Factors influencing health status and contact with health services	27,648 (72.6)	1,078,146 (56.0)	0.353	27,645 (72.6)	28,118 (73.9)	0.028
Comorbidities						
Essential (primary) hypertension	20,612 (54.1)	736,523 (38.2)	0.323	20,610 (54.1)	20,645 (54.2)	0.002
Dyslipidemia	16,920 (44.4)	620,052 (32.2)	0.254	16,919 (44.4)	16,879 (44.3)	0.002
Overweight and obesity	15,676 (41.2)	475,298 (24.7)	0.357	15,673 (41.2)	15,816 (41.6)	0.008
Neoplasms	11,759 (30.9)	343,283 (17.8)	0.308	11,758 (30.9)	12,043 (31.6)	0.016
Diabetes mellitus	10,383 (27.3)	316,704 (16.4)	0.264	10,381 (27.3)	10,248 (26.9)	0.008
Vitamin D deficiency	8,959 (23.5)	160,640 (8.3)	0.424	8,957 (23.5)	8,892 (23.4)	0.004
Ischemic heart diseases	5,490 (14.4)	191,972 (10.0)	0.136	5,488 (14.4)	5,434 (14.3)	0.004
Nicotine dependence	3,748 (9.8)	151,210 (7.9)	0.070	3,748 (9.8)	3,694 (9.7)	0.005
Diseases of liver	3,533 (9.3)	90,606 (4.7)	0.180	3,530 (9.3)	3,435 (9.0)	0.009
Heart failure	2,981 (7.8)	75,585 (3.9)	0.167	2,978 (7.8)	2,931 (7.7)	0.005
Cerebrovascular diseases	2,624 (6.9)	84,260 (4.4)	0.109	2,624 (6.9)	2,570 (6.8)	0.006
Chronic kidney disease (CKD)	2,329 (6.1)	52,442 (2.7)	0.166	2,328 (6.1)	2,258 (5.9)	0.008
Acute kidney failure	1,366 (3.6)	25,381 (1.3)	0.147	1,363 (3.6)	1,269 (3.3)	0.014
Alcohol related disorders	1,016 (2.7)	34,007 (1.8)	0.061	1,015 (2.7)	1,020 (2.7)	0.001
Malnutrition	510 (1.3)	5,210 (0.3)	0.120	507 (1.3)	458 (1.2)	0.012
Laboratory data						
Albumin ≥3.5 g/dL	24,848 (65.3)	843,427 (43.8)	0.442	24,846 (65.3)	25,441 (66.8)	0.033
eGFR >60 mL/min/1.73m^2^	25,369 (66.6)	922,141 (47.9)	0.386	25,366 (66.6)	25,851 (67.9)	0.027
Hemoglobin A1c > 7%	4,509 (11.8)	138,077 (7.2)	0.160	4,508 (11.8)	4,279 (11.2)	0.019
Medication						
Iron preparations	9,033 (23.7)	37,265 (1.9)	0.689	9,030 (23.7)	8,604 (22.6)	0.027
ACE inhibitors	7,549 (19.8)	299,484 (15.5)	0.112	7,548 (19.8)	7,390 (19.4)	0.010
Biguanides	6,270 (16.5)	194,832 (10.1)	0.188	6,270 (16.5)	6,118 (16.1)	0.011
Angiotensin II inhibitor	5,851 (15.4)	216,383 (11.2)	0.122	5,851 (15.4)	5,759 (15.1)	0.007
Insulins and analogues	4,973 (13.1)	132,896 (6.9)	0.207	4,970 (13.1)	4,828 (12.7)	0.011
GLP-1 analogues	1709 (4.5)	45,916 (2.4)	0.116	1709 (4.5)	1,626 (4.3)	0.011
SGLT2 inhibitors	890 (2.3)	28,329 (1.5)	0.063	890 (2.3)	847 (2.2)	0.008

IDA, iron deficiency anemia; data are presented as mean ± standard deviation for continuous variables and n (%) for categorical variables. BMI, body mass index; SMD, standardized mean difference; eGFR, estimated glomerular filtration rate; GLP-1, Glucagon-like peptide-1; SGLT2, Sodium-glucose co-transporter 2; †SMD values <0.1 indicate adequate balance between groups after matching.

### 3.2 Association between iron deficiency anemia and renal function decline at 5-year and 7-year follow-up

**Figure 2:**
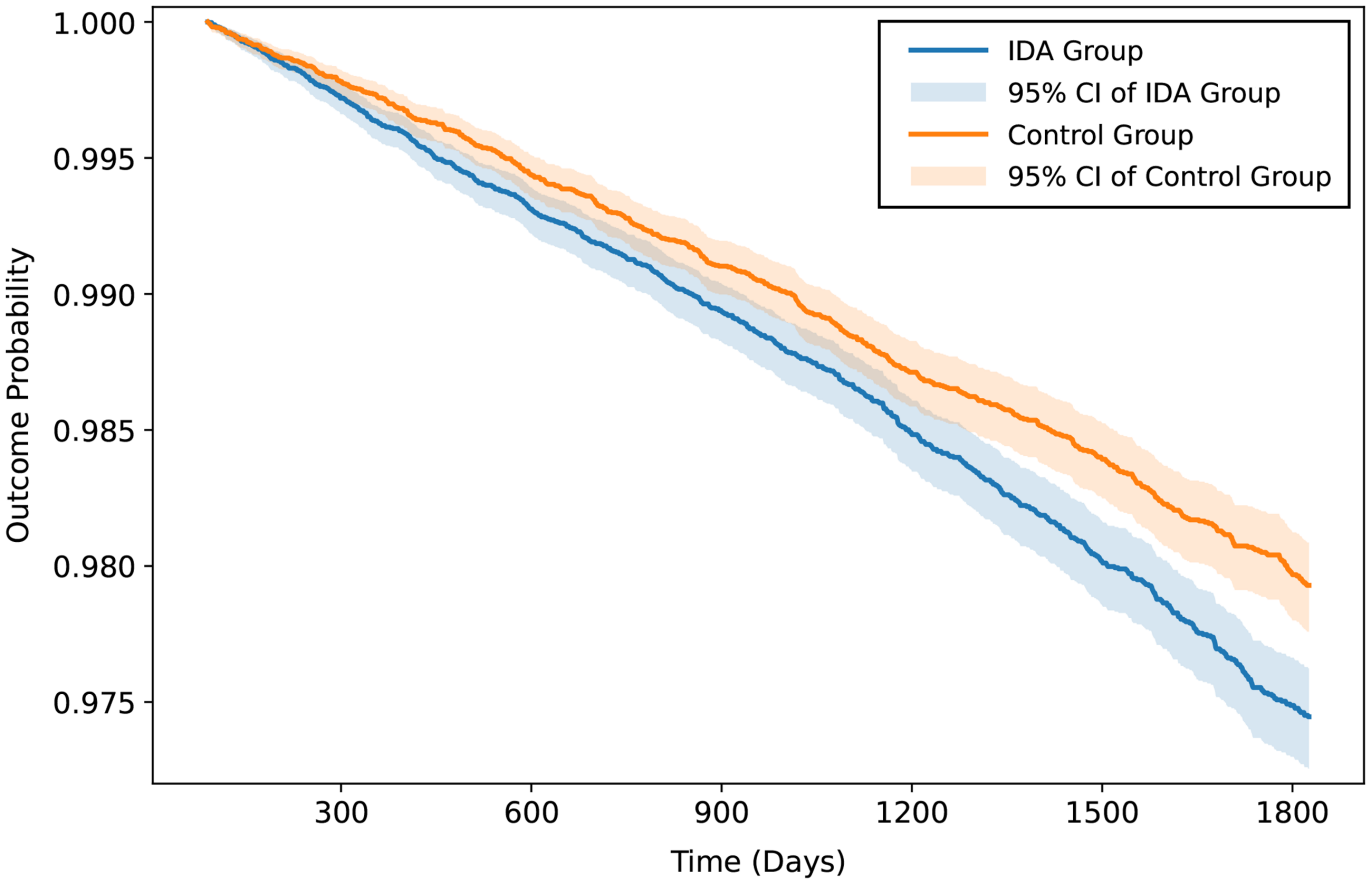
Cumulative incidence of composite renal function decline at 5-year follow-up among patients with obstructive sleep apnea (OSA) with and without iron deficiency anemia (IDA). At 5 years, composite renal function decline occurred in 757 patients (2.0%) with IDA versus 626 (1.6%) in the control group [hazard ratio (HR), 1.23; 95% confidence interval (CI), 1.10–1.37; *p* < 0.001]. Composite renal function decline was defined as progression to stage 4–5 chronic kidney disease, end-stage renal disease, or initiation of hemodialysis.

The mean follow-up period was 1,471 days in the IDA group and 1,487 days in the control group. At 5 years, composite renal function decline occurred in 757 patients (2.0%) with IDA vs. 626 (1.6%) in the control group (HR, 1.23; 95% CI, 1.10–1.37; *p* < 0.001) (Table 2) (Figure 2). AKI developed in 3944 patients (10.7%) with IDA vs. 3,286 (8.6%) in controls (HR, 1.22; 95% CI, 1.17–1.28; *p* < 0.001). IDA was also associated with increased cumulative incidence of pulmonary hypertension (4.5% vs. 3.7%; HR, 1.22; 95% CI, 1.14–1.31; *p* < 0.001), all-cause mortality (6.4% vs. 5.0%; HR, 1.29; 95% CI, 1.21–1.37; *p* < 0.001), and ICU admission (6.9% vs. 6.0%; HR, 1.17; 95% CI, 1.11–1.24; *p* < 0.001). At the 7-year follow-up, associations persisted for composite renal function decline (2.5% vs. 2.1%; HR, 1.23; 95% CI, 1.12–1.35; *p* < 0.001) and all secondary outcomes (Table 3).

**Table 2:** Association between iron deficiency anemia and adverse outcomes at 5-year follow-up.

Outcomes	IDA group	Control group	HR (95% CI)	*p*-value	ARD	NNH
	(*n* = 38,064)	(*n* = 38,064)				
	Events (%)	Events (%)				
Composite renal function decline	757 (2.0%)	626 (1.6%)	1.23 (1.10–1.37)	<0.001	0.4%	250
AKI	3,944 (10.7%)	3,286 (8.6%)	1.22 (1.17–1.28)	<0.001	2.1%	48
Pulmonary hypertension	1701 (4.5%)	1,414 (3.7%)	1.22 (1.14–1.31)	<0.001	0.8%	125
Mortality	2,442 (6.4%)	1921 (5.0%)	1.29 (1.21–1.37)	<0.001	1.4%	71
ICU admission	2,643 (6.9%)	2,285 (6.0%)	1.17 (1.11–1.24)	<0.001	0.9%	111

Values are expressed as event counts (percentages) unless otherwise indicated. HR, hazard ratio; CI, confidence interval; IDA, iron deficiency anemia; AKI, acute kidney injury; ICU, intensive care unit; ARD, absolute risk difference; NNH, number needed to harm; Composite renal function decline was defined as progression to stage 4–5 chronic kidney disease, end-stage renal disease, or hemodialysis initiation.

**Table 3:** Association between iron deficiency anemia and adverse outcomes at 7-year follow-up.

Outcomes	IDA group	Control group	HR (95% CI)	*p*-value
	(*n* = 38,064)	(*n* = 38,064)		
	Events (%)	Events (%)		
Composite renal function decline	951 (2.5%)	798 (2.1%)	1.23 (1.12–1.35)	<0.001
AKI	4,610 (12.1%)	3,950 (10.4%)	1.21 (1.16–1.26)	<0.001
Pulmonary hypertension	2022 (5.3%)	1728 (4.5%)	1.20 (1.13–1.28)	<0.001
Mortality	2,967 (7.8%)	2,435 (6.4%)	1.25 (1.19–1.32)	<0.001
ICU admission	3,166 (8.3%)	2,775 (7.3%)	1.17 (1.12–1.24)	<0.001

Values are expressed as event counts (percentages) unless otherwise indicated. HR, hazard ratio; CI, confidence interval; IDA, iron deficiency anemia; AKI, acute kidney injury; ICU, intensive care unit; Composite renal function decline was defined as progression to stage 4–5 chronic kidney disease, end-stage renal disease, or hemodialysis initiation.

### 3.3 Sensitivity analyses

In Model I (patients diagnosed 2016–2020; *n* = 28,214 matched pairs), IDA remained associated with composite renal function decline (HR, 1.28; 95% CI, 1.14–1.44; *p* < 0.001), AKI (HR, 1.19; 95% CI, 1.13–1.25; *p* < 0.001), pulmonary hypertension (HR, 1.20; 95% CI, 1.11–1.29; *p* < 0.001), mortality (HR, 1.25; 95% CI, 1.17–1.33; *p* < 0.001), and ICU admission (HR, 1.19; 95% CI, 1.12–1.26; *p* < 0.001) (Table 4). Model II excluded patients with history of CKD (*n* = 36,918 matched pairs). Among patients with preserved baseline renal function, associations persisted for composite renal function decline (HR, 1.20; 95% CI, 1.06–1.37; *p* = 0.005) and all secondary outcomes.

**Table 4:** Sensitivity analyses of the association between iron deficiency anemia and clinical outcomes at 5-year follow-up.

Outcomes	Model I (*n* = 28,214)		Model II (*n* = 36,918)	
	HR (95% CI)	*p*-value	HR (95% CI)	*p*-value
Composite renal function decline	1.28 (1.14–1.44)	<0.001	1.20 (1.06–1.37)	0.005
AKI	1.19 (1.13–1.25)	<0.001	1.18 (1.12–1.24)	<0.001
Pulmonary hypertension	1.20 (1.11–1.29)	<0.001	1.26 (1.17–1.36)	<0.001
Mortality	1.25 (1.17–1.33)	<0.001	1.26 (1.18–1.34)	<0.001
ICU admission	1.19 (1.12–1.26)	<0.001	1.15 (1.08–1.21)	<0.001

HR, hazard ratio; CI, confidence interval; AKI, acute kidney injury; ICU, intensive care unit; IDA, iron deficiency anemia; Composite renal function decline was defined as progression to stage 4–5 chronic kidney disease, end-stage renal disease, or hemodialysis initiation; Model I included patients diagnosed between 2016 and 2020; Model II excluded patients with baseline chronic kidney disease.

### 3.4 Subgroup analysis

Subgroup analyses showed consistent associations between IDA and composite renal function decline across demographic and clinical characteristics at 5 years (Table 5). No significant interactions were detected (all *p* values for subgroup differences >0.05). HRs ranged from 1.13 to 1.46 across subgroups defined by sex, age, hypertension, obesity, cardiovascular disease, and diabetes mellitus.

**Table 5:** Subgroup analyses of the association between iron deficiency anemia and composite renal function decline at 5-year follow-up.

Subgroup analysis	HR (95% CI)	*p*-value	*p*-value for Subgroup difference
Sex			
Male	1.13 (0.97–1.31)	0.129	Reference
Female	1.25 (1.08–1.45)	0.002	0.349
Age			
18–50 years	1.46 (0.99–2.15)	0.055	Reference
>50 years	1.26 (1.13–1.40)	<0.001	0.510
Hypertension			
Yes	1.13 (1.01–1.26)	0.035	Reference
No	1.22 (0.97–1.55)	0.092	0.576
Obesity			
Yes	1.26 (1.08–1.48)	0.003	Reference
No	1.29 (1.12–1.48)	<0.001	0.527
Cardiovascular disease			
Yes	1.16 (1.01–1.34)	0.043	Reference
No	1.25 (1.08–1.45)	0.003	0.477
DM			
Yes	1.31 (1.15–1.49)	<0.001	Reference
No	1.26 (1.06–1.50)	0.010	0.725

HR, hazard ratio; CI, confidence interval; DM, diabetes mellitus.

## 4 Discussion

This multi-institutional cohort study demonstrated a significant association between IDA and adverse renal outcomes in adults with OSA. After propensity score matching of 38,064 paired patients, IDA was associated with a 23% higher cumulative incidence of composite renal function decline at both 5-year and 7-year follow-ups. These associations persisted across multiple sensitivity analyses and remained consistent in patients with preserved baseline renal function. Secondary outcomes, including AKI, pulmonary hypertension, all-cause mortality, and ICU admission, were all significantly associated with IDA. Subgroup analyses revealed uniform associations across demographic and clinical characteristics, with no significant effect modification detected by sex, age, hypertension status, obesity, cardiovascular disease, or diabetes mellitus.

Current evidence has consistently demonstrated that OSA is linked to an elevated risk of developing CKD. Lee et al. (14) observed a 1.94-fold higher incidence of CKD among patients with sleep apnea. Similarly, a systematic review and meta-analysis encompassing 18 studies reported a pooled odds ratio of 1.77, confirming a robust association between OSA and CKD (25). However, the present study represents the first large-scale investigation to specifically examine the relationship between preexisting IDA and subsequent renal function decline in patients with OSA. In this study, patients with IDA exhibited a 23% higher risk of composite renal function decline, indicating that IDA may be associated with an additional risk of kidney disease progression beyond the known effects of OSA. This finding extends the current understanding by identifying a potentially modifiable nutritional factor that may influence renal outcomes in this vulnerable population. The persistence of these associations in sensitivity analyses strengthens the robustness of these observations and suggests that IDA may be associated with renal injury, even in individuals with preserved baseline kidney function.

The observed association between IDA and AKI in our cohort provides additional evidence linking IDA to renal injury in patients with OSA. Prior studies have established that OSA may predispose individuals to AKI through intermittent hypoxia, sympathetic activation, and endothelial injury. For example, Chuang et al. (26) reported that OSA severity correlated positively with multiple serum markers of AKI, including cystatin C, neutrophil gelatinase-associated lipocalin (NGAL), and interleukin-18, whereas continuous positive airway pressure therapy reduced albuminuria and IL-18 levels. Similarly, Nowicki et al. (27) demonstrated overnight elevations in urinary NGAL and kidney injury molecule-1 following apneic episodes, suggesting subclinical renal injury. Furthermore, Dou et al. (28) identified OSA as an independent risk factor for AKI among critically ill patients. Building on this evidence, the present study extends our understanding by examining the association between pre-existing IDA and subsequent AKI within an OSA population. The finding that IDA in patients with OSA is associated with both chronic renal function decline and AKI strengthens the hypothesis that IDA may be associated with multiple forms of kidney injury in this population. While previous studies have primarily focused on anemia as a predictor of AKI in surgical populations (29–31), the present study demonstrates this association specifically in the OSA population.

Iron deficiency commonly coexists with pulmonary hypertension, with a high prevalence reported across various subtypes of pulmonary hypertension (32). Experimental studies have shown that iron deficiency can promote pulmonary vascular remodeling and elevate pulmonary arterial pressure, suggesting a mechanistic link between disrupted iron homeostasis and the development of pulmonary hypertension (33, 34). However, despite these preclinical findings, clinical evidence directly confirming a causal relationship between iron deficiency and pulmonary hypertension remains limited (35, 36). Our study appears to be the first large-scale analysis to specifically evaluate the association between pre-existing IDA and incident pulmonary hypertension within the OSA population. The clinical importance of this finding is underscored by the serious nature of pulmonary hypertension as a complication. Pulmonary hypertension is characterized by elevated pulmonary artery pressure and progressive pulmonary vascular remodeling, which can lead to right ventricular dysfunction and heart failure if left untreated (37, 38). In patients with OSA who already face increased cardiovascular risks, the development of pulmonary hypertension represents a particularly concerning complication that may substantially impact prognosis. These findings broaden the current understanding of pulmonary vascular disease in OSA and highlight IDA as a potential modifiable risk factor.

These findings have important implications for the management of patients with OSA. IDA may serve as a marker identifying individuals at heightened risk for renal function decline and pulmonary vascular complications, warranting closer monitoring of the kidney and cardiovascular status. The observed associations with both chronic and AKI suggest that evaluation of IDA may be valuable across the continuum of OSA care, including during acute illnesses or perioperative periods. Although the current OSA guidelines rarely address nutritional deficiencies, these results support the incorporation of routine iron assessment into clinical practice. Whether correcting iron deficiency through supplementation or treatment of the underlying causes can improve renal or pulmonary outcomes remains to be determined in future prospective studies. The additional associations between all-cause mortality and ICU admission highlight the broader clinical relevance of IDA and the need for comprehensive risk management in this population.

In the current study, several limitations warrant consideration. First, the retrospective observational design precludes definitive conclusions regarding causality, and residual confounding from unmeasured variables remains possible. Although a broad range of demographic and clinical variables were included in the propensity score matching model, specific autoimmune and systemic inflammatory diseases could not be comprehensively ascertained or modeled in the TriNetX database. Residual confounding related to these immune-mediated conditions, which may influence both anemia and renal outcomes, therefore cannot be excluded. Second, information regarding iron supplementation during follow-up was not systematically captured, potentially attenuating the observed associations if some patients received treatment. Detailed iron parameters, including serum ferritin, transferrin saturation, and markers distinguishing absolute from functional iron deficiency, were not systematically available. Third, both OSA and IDA were identified using ICD-10 diagnostic codes without direct clinical validation; the lack of polysomnography data for OSA and laboratory confirmation of iron indices or hemoglobin levels for IDA introduces a risk of diagnostic misclassification, including miscoding of anemia subtypes and inter-institutional variability in coding practices, which may bias exposure and outcome ascertainment and attenuate the observed associations. In addition, disease severity could not be assessed, as information on OSA severity (e.g., apnea–hypopnea index) and treatment adherence (e.g., continuous positive airway pressure use) was unavailable, precluding evaluation of dose–response relationships despite the known link between OSA severity and renal outcomes. As a result, we were unable to incorporate these factors into adjustment, stratified, or sensitivity analyses, and potential effect modification by OSA severity cannot be excluded. These limitations are inherent to large-scale electronic health record–based studies and highlight the need for prospective investigations incorporating polysomnography, detailed iron parameters, and longitudinal renal biomarkers to validate and extend these findings. Fourth, serial kidney function measurements were not systematically obtained, which precluded detailed trajectory analyses. Finally, mechanistic biomarkers, including inflammatory markers and oxidative stress parameters, were not consistently available.

## 5 Conclusion

This study demonstrated significant associations between IDA and increased risk of renal function decline, AKI, and pulmonary hypertension development in patients with OSA. These relationships persist across multiple analytical approaches. Future prospective investigations incorporating serial measurements of iron status, mechanistic biomarkers, and treatment adherence assessment are needed to clarify the temporal relationships. Randomized controlled trials evaluating whether interventions targeting iron deficiency favorably influence renal and pulmonary vascular outcomes in patients with OSA would provide evidence for potential therapeutic strategies.

## Data Availability

The raw data supporting the conclusions of this article will be made available by the authors, without undue reservation.
