# Supplementary material for: Iron deficiency anemia is associated with renal function decline in obstructive sleep apnea: a multi-institutional cohort study
Source: Front Nutr. 2026 Feb 12;13:1743873. doi: 10.3389/fnut.2026.1743873 (PMC12935634; doi:10.3389/fnut.2026.1743873)

**Supplemental Table 1.** Codes Used for Cohort Definition, Outcomes, and Propensity Score Matching

| Category | Variable | Definition | Codes |
| --- | --- | --- | --- |
| Inclusion criteria | Obstructive sleep apnea (OSA) | Diagnosis of OSA (adult or pediatric) | G47.33 |
|  | Iron deficiency anemia (IDA) | Diagnosis of iron deficiency anemia prior to index date | D50 |
|  | Age | Adults ≥18 years at index date | — |
| Exclusion criteria | Non-IDA anemias | Vitamin B12 deficiency anemia | D51 |
|  |  | Folate deficiency anemia | D52 |
|  |  | Other nutritional anemias | D53 |
|  |  | Anemia in chronic disease | D63 |
|  |  | Other/unspecified anemias | D64 |
|  | Advanced CKD | CKD stage 4 | N18.4 |
|  |  | CKD stage 5 | N18.5 |
|  |  | End-stage renal disease | N18.6 |
|  | Dialysis dependence | Dialysis dependence | Z99.2 |
|  | Hemodialysis | Hemodialysis procedures | 1012752 |
|  |  | Hemodialysis | 39.95 |
|  |  | Hemodialysis | 302497006 |
|  | Severe renal dysfunction | eGFR ≤30 mL/min/1.73 m² | 98979-8 |
|  | Pulmonary hypertension | Primary pulmonary hypertension | I27.0 |
|  |  | Other secondary pulmonary hypertension | I27.2 |
|  | Major surgery | Bariatric surgery procedures | 1007385 |
|  |  | Nephrectomy (any approach) | 1014157 |
|  |  | Partial nephrectomy | 50240 |
|  | Kidney disorders | Cystic kidney disease | Q61 |
|  |  | Kidney transplant status | Z94.0 |
|  | Bariatric status | History of bariatric surgery | Z98.84 |
| Primary outcome | Composite renal function decline | CKD stage 4–5, ESRD, or hemodialysis initiation | N18.4, N18.5, N18.6, Z99.2; CPT 1012752; SNOMED 302497006; ICD-9-CM 39.95 |
| Secondary outcomes | Acute kidney injury (AKI) | Acute kidney failure | N17 |
|  | Pulmonary hypertension | Other secondary pulmonary hypertension | I27.2 |
|  | ICU admission | Critical care services | 1013729 |
|  | Mortality | Death or ill-defined cause of death | R99; Deceased |
| Variables for matching | Demographics | Age, sex, race | — |
|  | Cardiometabolic diseases | Hypertension | I10 |
|  |  | Diabetes mellitus | E08–E13 |
|  |  | Dyslipidemia | E78 |
|  |  | Obesity / overweight | E66 |
|  | Cardiovascular disease | Ischemic heart disease | I20–I25 |
|  |  | Heart failure | I50 |
|  | Cerebrovascular disease | Stroke and related disorders | I60–I69 |
|  | Renal disease | Chronic kidney disease (any stage) | N18 |
|  | Liver disease | Chronic liver diseases | K70–K77 |
|  | Nutritional status | Malnutrition | E40–E46 |
|  |  | Vitamin D deficiency | E55 |
|  | Substance use | Nicotine dependence | F17 |
|  |  | Alcohol-related disorders | F10 |
|  | Medications | ACE inhibitors | CV800 |
|  |  | Angiotensin II receptor blockers | CV805 |
|  |  | Insulin and analogues | A10A |
|  |  | Biguanides | A10BA |
|  |  | GLP-1 receptor agonists | A10BJ |
|  |  | SGLT2 inhibitors | A10BK |
|  |  | Iron preparations | B03A |
|  | Laboratory variables | Albumin | 9045 |
|  |  | Hemoglobin A1c | 9037 |
|  |  | Estimated GFR | 62238-1 |

**Supplemental Figure 1. Love plot showing standardized mean differences of covariates before and after propensity score matching**


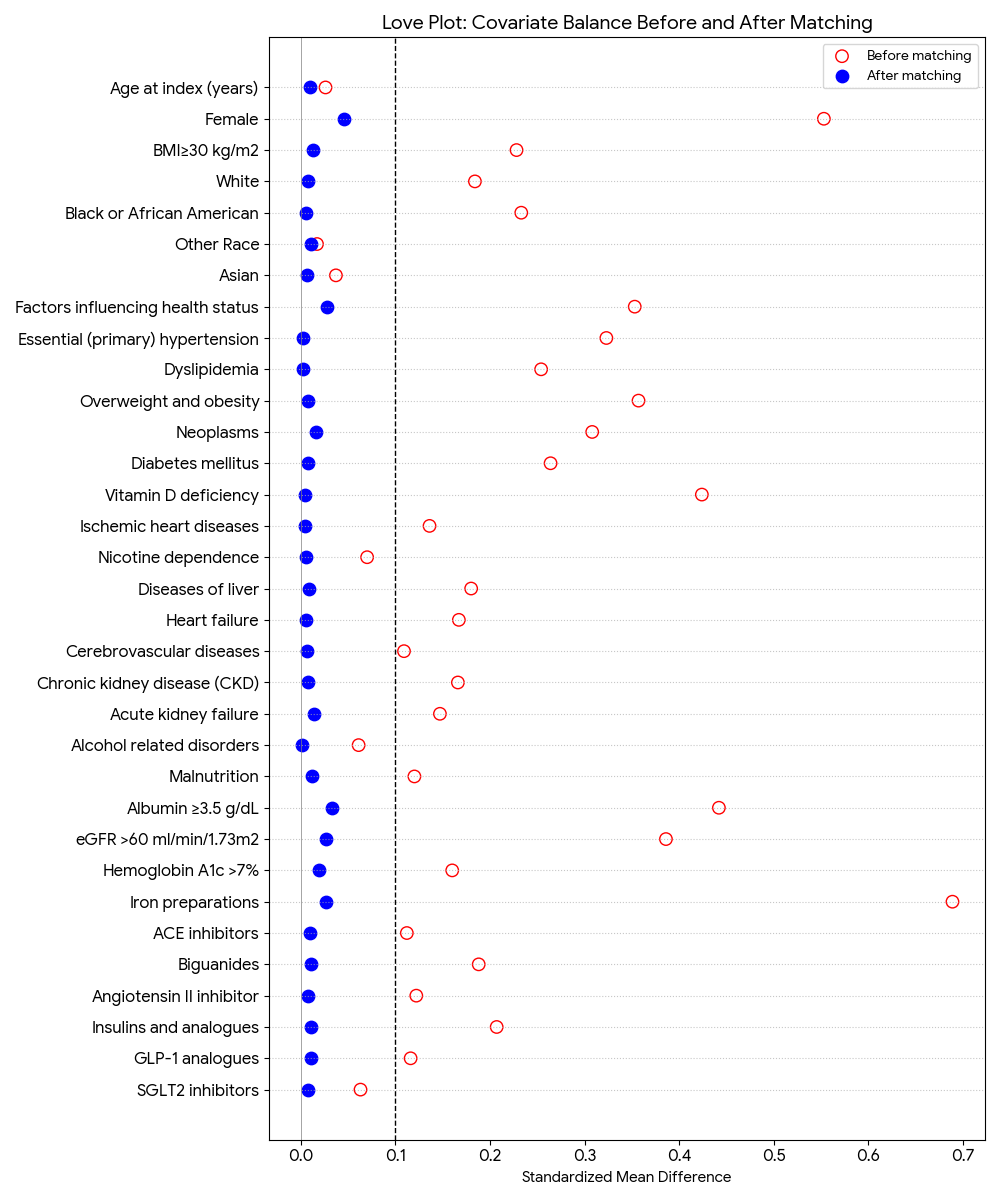

Supplement: Supplementary file 1 [file Table_1.docx]
